# Prevalence, Aetiology, and Antimicrobial Susceptibility Patterns of Urinary Tract Infection Amongst Children Admitted at Kilimanjaro Christian Medical Centre, Moshi, Tanzania

**DOI:** 10.24248/EAHRJ-D-16-00341

**Published:** 2017-03-01

**Authors:** Joshua G Gidabayda, Rune Philemon, Mohammed S Abdallah, Aliasgher M Saajan, Theresia Temu, Ipyana Kunjumu, Blandina T Mmbaga, Levina J Msuya

**Affiliations:** a Kilimanjaro Christian Medical Centre, Moshi, Tanzania; b Kilimanjaro Christian Medical University College, Moshi, Tanzania; c Kilimanjaro Clinical Research Institute, Moshi, Tanzania

## Abstract

**Background:**

Urinary tract infections (UTIs) in the paediatric population are well recognised as a cause of acute morbidity and chronic medical conditions, such as hypertension and renal insufficiency later in adulthood. Although antimicrobial treatment of UTIs is simple, the disease is still largely misdiagnosed and mismanaged. Moreover, increasing resistance to conventional antimicrobials is eroding the success of empiric therapy.

**Objective:**

To determine prevalence, aetiological agents, and antimicrobial sensitivity patterns of UTIs amongst children admitted at Kilimanjaro Christian Medical Centre (KCMC).

**Methodology:**

A cross-sectional, hospital-based study was conducted at the KCMC Department of Paediatrics and Child Health between December 2013 and April 2014. All children ages 2 months to 14 years who were admitted in the paediatric ward during the study period and fulfilled study criteria were enrolled. Data were collected by structured questionnaires. A urine dipstick test was done to detect the presence of nitrites and leucocytes, and to perform microscopic analysis of leucocytes and bacteria. All positive cases with the urine dipstick were cultured to determine bacterial species and antimicrobial susceptibility. Urine culture is considered the gold standard to confirm UTI.

**Results:**

A total of 343 children enrolled in the study. Of these, 208 (60.6%) were male and 135 (39.4%) were female. The urine dipstick test was positive for leucocyte esterase and nitrate in 87 (25.4%) and 33 (9.6%), respectively, and urine microscopy showed leucocytes and bacteria by microscope in 38 (11.1%) and 24 (7.0%) samples, respectively. UTI was confirmed by culture in 11.4% (39/343) of the samples. Female children and children less than 24 months old had a higher prevalence of UTI (17% and 15.8%, respectively). Female sex (odds ratio [OR] 2.46, 95% confidence interval [CI], 1.25–4.86), presence of leucocytes esterase (OR 32.20, 95% CI, 12.03–86.19), and nitrate in urine dipstick (OR 5.87, 95% CI, 3.44–3.65) were associated with UTI. Leucocyte esterase, nitrite, microscopic leucocyte, and bacteria were positive in 34 (87.2%), 24 (61.5%), 30 (78.9%), and 23 (59%) samples, respectively, using culture as a gold standard. Antimicrobial sensitivity of nitrites, leucocyte esterase, microscopic leucocyte, and bacteria was 38.1%, 87.2%, 97.4%, and 59.0%, respectively, and specificity was 94.1%, 82.6%, 82.2%, and 99.7%. The most common bacterial species isolated were *Escherichia coli* 46.2% (18/39) and *Klebsiella pneumoniae* 30.8% (12/39); both exhibited low susceptibility to ampicillin, co-trimoxazole, and clindamycin, but they were susceptible to ciprofloxacin, nalidixic acid, and ceftazidime.

**Conclusions:**

UTIs are common conditions affecting children admitted at KCMC. The prevalence is higher in infants and children younger than 24 months. *E coli* and *K pneumoniae* were the most common isolated organisms with low susceptibility in commonly used antibiotics. Antimicrobials, such as ciprofloxacin, ceftriaxone, and gentamicin, are more likely to be successful for empirical treatment of UTIs.

## INTRODUCTION

Urinary tract infections (UTIs) are amongst the most common infections in children, occurring in as many as 5% of girls and 1%–2% of boys.^1^ It has been estimated that globally, symptomatic UTIs result in as many as 7 million visits to outpatient clinics, 1 million visits to emergency departments, and 100,000 hospitalisations annually.^2^ UTIs occur in 10% of all febrile children, 13.6% of febrile infants, and 7% of febrile newborns.^3^ UTIs have great clinical significance due to their acute complications, such as mortality in infants, as well as chronic complications, such as hypertension and chronic renal failure following chronic pyelonephritis. Pyelonephritis accounts for 7%–30% of end-stage renal failure in some countries.^4^ The early phase of tissue invasion by microorganisms is the critical determinant in the pathogenesis of kidney lesions following a UTI; therefore, early diagnosis with prompt and effective antimicrobial treatment for acute renal infection can prevent or significantly inhibit the development of renal damage.^1^

The prevalence of UTIs is roughly 9% in developed countries and 45% in developing countries of children seeking medical attention for any cause.^4,5^ The few studies in Tanzania have shown a prevalence range from 16.7% to 39.7%.^6–8^ A variety of bacteria are thought to cause UTIs, in particular *Escherichia coli, Klebsiella pneumoniae, Proteus mirabilis, Staphylococcus aureus, Pseudomonas aeruginosa, and Acinetobacter baumannii.*^9,10^ This wide range of causative agents suggests that many UTIs may be opportunistic infections.

Although UTIs generally respond well to antimicrobial treatment, on a global basis there is increasing resistance amongst organisms that cause UTIs. There is a need to gain local knowledge about the types of pathogens responsible for UTIs and know their resistance patterns to help clinicians choose effective empiric treatment.^11^ Moreover, the community needs to know whether we are exhausting treatment options and whether more effort is required with prevention strategies. Our immediate aim was to determine which bacteria commonly cause UTIs in our locality and to gain knowledge on their antimicrobial susceptibilities.

## MATERIAL AND METHODS

### Study Area

The study was conducted at Kilimanjaro Christian Medical Centre (KCMC) at the Department of paediatric and Child Health. KCMC is a referral hospital for over 15 million people in the Northern Zone of Tanzania (Kilimanjaro, Tanga, Arusha, and Manyara). It has daily attendance of 500 outpatients, nearly 500 in-patients with 640 official bed capacity, 1,000 staff, and hundreds of visitors daily. As a consultant hospital, it receives children from other health facilities both within the catchment area and from other regions of Tanzania. The paediatric bed capacity is over 100 beds.

### Study Design

This was a cross-sectional, hospital-based study conducted from December 2013 to April 2014.

### Study Population

The study included children from 2 months to 14 years of age admitted to the general paediatric ward during the study period with or without symptoms of UTI. Children under 2 months of age and children whose parents and guardians refused to give consent were excluded from the study.

The prevalence of UTI, uropathogens, and antimicrobial sensitivity patterns were considered to be dependent variables whereby age, sex, antibiotics use, nutritional status, and duration of treatment were all independent variables.

### Sample Size and Sampling Technique

A required minimum sample size for this study was calculated using a standard formula for calculating sample size, N=[Z^2^P(1−P)]/E^2^. Taking the prevalence of UTI as 16.3%, as reported by Fredric and colleagues in 2013 at Muhimbili,^9^ the critical value Z=1.96, equivalent to a confidence interval (CI) level of 95% with a minimum tolerable error of 5%. The minimum sample size was estimated to be 210. Convenient sample technique was considered. Children were assessed during admission, and parents or caretakers received an explanation about the study and signed informed consent forms if the child fulfilled inclusion criteria for enrolment and they agreed to participate. Children whose parents or caretakers were not ready to give consent received routine services.

### Data Collection

All children who fulfilled the inclusion criteria during admission and whose parents or caretakers signed the informed consent were included in the study. A standardised, structured questionnaire was used to gather demographic and clinical characteristics of children and a laboratory data sheet was used to collect laboratory information, which included urine dipstick and urine microscopic results, pathogen species, and antimicrobial susceptibility. Urine was collected by both clean-catch midstream for older children and urethral catheterisation for young children (<2 years). Two urine specimens were collected in 2 sterile urine containers. One specimen underwent spot testing using the dipstick method to determine the nitrite and leucocyte levels in the urine. For specimens that had any positive results on the urine dip-stick (leucocyte esterase and nitrite), the second specimen was sent to the laboratory for microbial identification, culture, and susceptibility testing.

### Laboratory Methods

Urine samples were processed within 1 hour of collection, and if any delay was anticipated, the specimen was stored at 4–8°C and analysed within 24 hours. Urine microscopy was performed to identify the presence of white blood cells and bacteria. Culture processing was performed by inoculation of urine into a cysteine-lactose electrolyte-deficient medium using a standard 1μL loop. Culture of each urine specimen prior to centrifugation was done quantitatively on 5% blood agar and MacConkey agar plates that were incubated aerobically at 37°C for 24 hours. Colonies were counted, mixed-growth cultures were interpreted as negative culture, and only the growth of single-uropathogen colonies of ≥10^5^ CFU/ml was interpreted as a positive culture. Bacterial colonies on solid agar were then identified based on characteristic morphology and Gram stain appearance. All Gram-negative bacteria were identified using API 20E strips (bioMérieux Inc., Marcy-l'Étoile, France). Urine culture bacterial growth was used as a gold standard for confirmation of UTI.

### Drug Susceptibility Testing

Antimicrobial susceptibility testing was performed with selected commonly used antibiotics in our settings. These included ampicillin (10 μg), co-trimoxazole (30 μg), gentamicin (10 μg), amoxicillin-clavulanate (20/10 μg), nitrofurantoin, ciprofloxacin (5 μg), chloramphenicol (10 μg), nalidixic acid (10 μg), clindamycin (20 μg), and cephalosporins such as ceftriaxone (30 μg), cefazolin, and ceftazidime (30 μg). The testing was done by the Kirby-Bauer disc-diffusion method using Mueller-Hinton agar that was incubated for 18 to 24 hours at 37°C. Antimicrobial susceptibility was reported as resistant, intermediate, and sensitive according to Clinical Laboratory Standards Institute guidelines.^12^

### Statistical Analysis

Data were coded and entered into a computer using SPSS version 20 (SPSS Inc., Chicago, IL, USA) and summarised by frequency, median (interquartile range), and percentage. Odds ratios (OR) with a 95% confidence interval were used for reporting the association, with *P*<.05 considered significant. Furthermore, we compared the diagnostic tests with a gold standard method, and specificity, sensitivity, and positive and negative predictive values were estimated.

### Ethical Consideration

Ethical approval was secured from Kilimanjaro Christian Medical University College Research Ethics Committee. Children whose parents or caretakers who signed informed consent forms were included in the study. Those who did not give consent received routine service and care.

## RESULTS

### Demographic Characteristics of Study Participants

**FIGURE 1.**
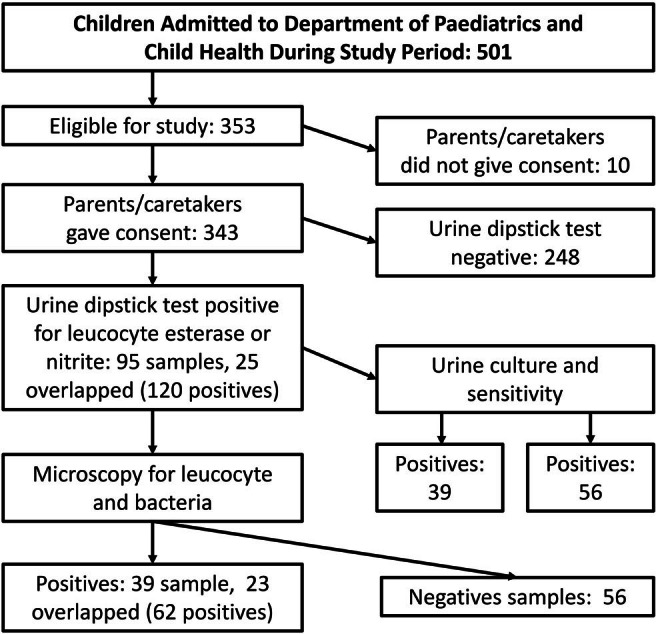
Number of Participants Enrolled in Study and Test Results

In total, 501 children were admitted to the Department of paediatric and Child Health during the study period; 148 children were below 2 months of age, and 10 did not give consent. Thus, 343 eligible children enrolled in the study as shown in Figure 1.

The median age at enrolment was 29 months (range: 2 months to 14 years). Male participants constituted a higher percentage (60.6%) compared to female participants. Table 1 shows the age and sex distribution of the study participants.

**TABLE 1.** Age and sex distribution of the participants (N=343)

Characteristics	n (%)
Age groups (years)	
≤1	152 (44.3)
2–4	87 (25.4)
5–7	39 (11.4)
8–10	29 (8.5)
11–13	36 (10.5)
Median (IQR)	42 (29–54)
Sex	
Male	208 (60.6)
Female	135 (39.4)

Abbreviation: IQR, interquartile range.

### Prevalence of UTIs

Of the 343 children enrolled in this study, the urine dipstick test was positive for leucocyte esterase and nitrate in 87 (25.4%) and 33 (9.6%) samples, respectively, and urine microscopy showed leucocytes and bacteria in 38 (11.1%) and 24 (7%) samples, respectively.

Urine culture was used as the confirmatory test for UTI; therefore, the prevalence of UTI was 11.4% (n=39). Children younger than 24 months had a higher prevalence of UTI (15.8%) compared to children ages 24–59 months and over 60 months (9.2% and 8.7%, respectively), and female children had a higher prevalence of UTI (17%) compared with their male counterparts (7.7%). High prevalence of UTI was observed in children with fever for about 2–10 days (17.7%), diarrhoea (16.7%), poor weight gain (16.7%), abdominal pain (16.2%), pain during urination (15.4%), and fever (14%) at baseline. Overall, 135 (39.4%) children were on antimicrobial treatment prior to enrolment, with observed lower prevalence of UTI compared with the group not exposed to antibiotics (8.1% vs. 13.5%) (Table 2). Only 86 (63.7%) knew the type of antibiotic used, with 47 (56%) being on ampicillin or amoxicillin alone, 12 (14.3%) on ampicillin in combination with gentamicin, and 10 (11.9%) on cephalosporin, with only (3.6%) exposed to co-trimoxazole.

**TABLE 2.** Prevalence of UTI Among Study Population by Characteristic (N=343)

Characteristics	Total	UTI No. (%)	OR (95% CI)	*P* Value
Age groups (months)				
<24	152	24 (15.8)	1.79 (0.79–4.05)	0.165
24–59	87	8 (9.2)	1.07 (0.40–2.90)	0.896
≥60	104	9 (8.7)	1.0	
Sex				
Male	208	16 (7.7)	1.0	
Female	135	23 (17.0)	2.46 (1.25–4.86)	0.008
Abdominal pain				
Yes	37	6 (16.2)	1.5 (0.69–3.44)	0.299
No	304	32 (10.5)	1.0	
Flank pain				
Yes^a^	5	1 (20.0)	1.78 (0.30–10.53)	0.455
No	338	38 (11.2)	1.0	
Vomiting				
Yes	108	13 (12.0)	1.09 (0.58–2.03)	0.792
No	235	26 (11.1)	1.0	
Diarrhoea				
Yes	54	9 (16.7)	1.59 (0.80–3.17)	0.188
No	287	30 (10.5)	1.0	
Fever				
Yes	179	25 (14.0)	1.64 (0.88–3.03)	0.114
No	164	14 (8.5)	1.0	
Duration of fever (n=179)				
0–2 days	51	9 (17.7)	1.49 (0.60–3.6)	0.823
3–7 days	119	15 (12.6)	1.0	0.897
7+ days	7	1 (14.3)	1.16 (0.13–10.28)	
Antimicrobial use				
Yes	135	11 (8.1)	1.75 (0.84–3.65)	0.13
No	208	28 (13.5)	1.0	
Painful urination				
Yes^a^	13	2 (15.4)	1.37 (0.0.37–5.89)	0.642
No	330	37 (11.2)	1.0	
Poor weight gain				
Yes	42	7 (16.7)	1.57 (0.74–3.32)	0.248
No	301	32 (10.6)	1.0	
Leucocyte esterase				
Positive	87	34 (39.1)	32.0 (12.03–86.18)	<0.001
Negative	256	5 (2.0)	1.0	
Nitrite				
Positive	33	15 (45.5)	5.87 (3.44–3.65)	<0.001
Negative	310	24 (7.7)	1.0	

Abbreviations: OR, odds ratio; UTI, urinary tract infection.

a The Fisher exact test was used, as some cells have fewer than 5 events.

Amongst the baseline characteristics, female sex (OR 2.46, 95% CI, 1.25–4.86), and presence of leucocytes esterase (OR 32.0, 95% CI, 12.03–86.18) or nitrate in urine (OR 5.87, 95% CI, 3.44–3.65) were the only factors associated with UTI. Although not statistically significant, young age had nearly twice increased odds of having UTI (OR 1.79, 95% CI, 0.79–4.05).

### Comparing Urine Dipstick and Microscopic Examination With Urine Culture

Of the children with UTI confirmed by urine culture (n=39), urine dipstick showed positive results for leucocyte esterase and nitrite in 34 (87.2%) and 15 (38.5%) samples, respectively, and 15.4% were positive for both leucocyte esterase and nitrite; urine microscopy showed positive results for leucocyte and bacteria in 30 (76.9%) and 23 (59%) samples, respectively. Antimicrobial sensitivity of nitrites, leucocyte esterase, microscopic leucocyte, and bacteria was 35.1%, 87.2%, 97.4%, and 59.0%, respectively, and specificity was 94.1%, 82.6%, 82.2%, and 99.7% (Table 3).

**TABLE 3.** Comparison of Urine Dipstick and Urine Microscopic Results With Urine Culture

	Urine Culture			
Tests	PPV (%)	NPV (%)	Sensitivity (%)	Specificity (%)
Urine dipstick				
Nitrite	45.5	92.3	38.5	94.1
Leucocyte esterase	39.1	98	87.2	82.6
Microscopy				
Leucocyte	41.3	99.6	97.4	82.2
Bacteria	95.8	95	59	99.7

Abbreviations: NPV, negative predictive value; PPV, positive predictive value.

### Isolated Microorganisms and Their Antimicrobial Susceptibly Patterns

**FIGURE 2.**
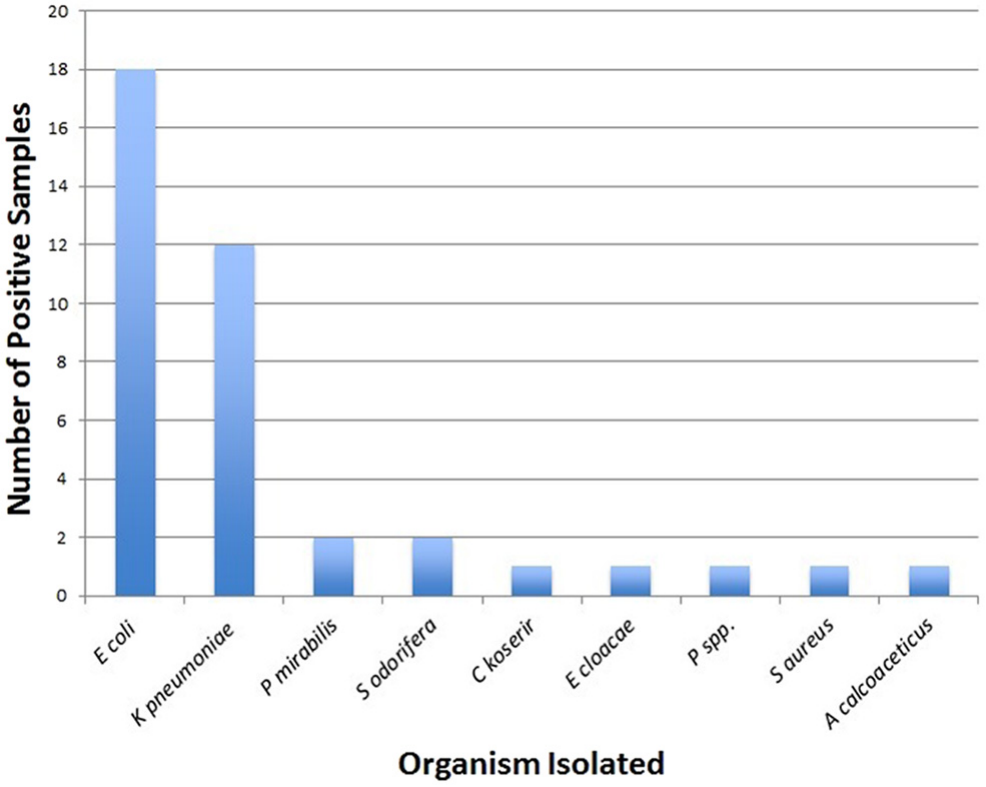
Number of Positive Samples and Isolated Organisms (n=39)

A total of 9 pathogenic bacteria were isolated from 39 positive cultures. *E coli* was the most common isolated pathogen in 18 (46.2%) children, followed by *K pneumoniae* in 12 (30.8%) children, and only 1 (2.6%) child had Gram-positive bacteria *S aureus* (Figure 2).

Isolated pathogens were tested for their antimicrobial susceptibility patterns with a total of 12 antibiotics. *E coli* isolates (n=18) were least susceptible to clindamycin (22.2%), co-trimoxazole (33.3%), and ampicillin (38.9%). *K pneumoniae* isolates (n=12) were highly susceptible to ciprofloxacin (75%), nalidixic acid (75%), and ceftazidime (66.7%). Table 4 shows the antimicrobial susceptibility patterns of the isolated organisms.

**TABLE 4.** Antimicrobial Susceptibility Patterns of the Isolated Organisms (N=39)

	Isolated Organism No. (%)								
Antibiotics	*E coli*	*A c*	*C k*	*E c*	*K p*	*P m*	*P s*	*S o*	*S a*
Ampicillin	7 (38.9)	0 (0.0)	0 (0.0)	0 (0.0)	1 (8.3)	0 (0.0)	0 (0.0)	1 (50.0)	1 (100.0)
Co-trimoxazole	6 (33.3)	1 (100.0)	1 (100.0)	0 (0.0)	3 (25.0)	1 (50.0)	0 (0.0)	1 (50.0)	0 (0.0)
Gentamicin	14 (77.8)	1 (100.0)	1 (100.0)	0 (0.0)	7 (58.3)	1 (50.0)	0 (0.0)	1 (50.0)	1 (100.0)
Chloramphenicol	15 (83.3)	0 (0.0)	1 (100.0)	1 (100.0)	9 (58.3)	1 (50.0)	0 (0.0)	2 (100.0)	1 (100.0)
Clindamycin	4 (22.2)	0 (0.0)	0 (0.0)	0 (0.0)	1 (8.3)	0 (0.0)	0 (0.0)	0 (0.0)	0 (0.0)
Amoxicillin-clavulanate	13 (72.2)	1 (100.0)	1 (100.0)	0 (0.0)	7 (58.3)	1 (50.0)	0 (0.0)	1 (50.0)	1 (100.0)
Nitrofurantoin	15 (83.3)	0 (0.0)	1 (100.0)	1 (100.0)	7 (58.3)	0 (0.0)	0 (0.0)	2 (100.0)	0 (0.0)
Ciprofloxacin	16 (88.9)	1 (100.0)	1 (100.0)	1 (100.0)	9 (75.0)	1 (50.0)	0 (0.0)	2 (100.0)	1 (100.0)
Ceftriaxone	15 (83.3)	0 (0.0)	1 (100.0)	1 (100.0)	6 (50.0)	2 (100.0)	0 (0.0)	2 (100.0)	1 (100.0)
Nalidixic acid	12 (66.7)	1 (100.0)	1 (100.0)	1 (100.0)	9 (75.0)	2 (100.0)	0 (0.0)	2 (100.0)	1 (100.0)
Cefazolin	15 (83.3)	0 (0.0)	1 (100.0)	1 (100.0)	5 (41.7)	1 (50.0)	0 (0.0)	2 (100.0)	0 (0.0)
Ceftazidime	14 (77.8)	1 (100.0)	1 (100.0)	0 (100.0)	8 (66.7)	2 (100.0)	1 (100.0)	2 (100.0)	1 (100.0)
**Total**	**18**	**1**	**1**	**1**	**12**	**2**	**1**	**2**	**1**

Abbreviations: *A c, Acinetobacter calcoaceticus; C k, Citrobacter koseri; E c, Enterobacter cloacae; K p, Klebsiella pneumoniae; P m, Proteus mirabilis; P s, Pseudomonas spp; S o, Serratia odorifera; S a, Staphylococci aureus.*

## DISCUSSION

This study on UTIs was done to determine the prevalence, aetiological agent, and antimicrobial susceptibility in children with UTIs admitted in a tertiary care hospital in northern Tanzania. Since we aimed to look at the overall prevalence in the paediatric ward, we did not restrict the study to patients with fever, UTI symptoms, or not on antibiotics; and we used urine culture as a gold standard for diagnosis of UTI. The prevalence of UTIs in this population was 11.4%, a finding that is comparable to previous studies done in both developed and developing countries. ^1,13,14^ Our findings are similar to a study done at Muhimbili National Hospital by Fredrick and colleagues, where a UTI prevalence of 16.8% was found amongst febrile children.^8^ The prevalence of UTI in our study population was lower, however, than the 20.3% reported by Msaki and colleagues in Tanzania^7^ and a study done in Bugando Medical Centre that reported a prevalence of 39.7%.^8^ The higher prevalence in Msaki's study might be because their study was conducted in a primary health facility and included children with fever only, whereas our study was conducted at a tertiary health facility and included all children who were symptomatic and asymptomatic. Of the 135 (39.4%) children in our study who were on antibiotics prior to admission, the majority were on ampicillin or amoxicillin (56%), ampicillin and gentamicin (14.3%), and cephalosporin (11.9%). Previous exposure to antibiotics might be the cause of the lower susceptibility to ampicillin.

In this study, positive leucocyte esterase and nitrite were the only risk factors of UTI (OR 32.2 and 5.87, respectively). UTI may present with nonspecific symptoms in children; however, abdominal pain, flank pain, vomiting, diarrhoea, duration of fever, antibiotics use, painful urination, and poor weight gain were not associated with UTI. Similar findings have been observed in Nigeria, in keeping with nonspecific symptoms of UTI in children.^13^ Children enrolled were of different ages, with the majority being younger, which may underscore the presence of some symptoms such as abdominal pain and painful micturition. Vomiting alone is unlikely to be a sign of UTI, as the observed risk was only 9%. However, it should not be ignored as one of the signs of UTI.

Female children had a higher prevalence of UTI compared with males, with a female-to-male ratio of 2:1. This result is consistent with previous studies in Gaza, Tanzania, and India with female-to-male ratios of 4:1, 1.7:1, and 1.5:1, respectively.^7,9,10^ A possible explanation for this difference is that females have anatomical and physical features (eg, short urethra) that predispose them to ascending infection. Therefore, the significant twofold increased risk of UTI in female children shows that one should highly suspect UTI in female children presenting with specific or nonspecific symptoms. Dipstick tests (for nitrites and leucocyte esterase) and microscopic examination (for leucocyte and bacteria) in urine can be used as screening tools for UTI since they have shown a high specificity and negative predictive value; however, the observed low antimicrobial sensitivity for nitrites and leucocytes indicate a need for a confirmatory test like urine culture, as used in this study. Furthermore, positive urine dipstick results for nitrites and leucocytes esterase, as observed in other studies, should prompt clinicians to consider a diagnosis of UTI in children,^8^ especially when culture testing is not available.

Infants and children younger than 2 years old tend to have high chances of acquiring UTIs. In this study, children under the age of 2 had a higher prevalence of UTIs, similar to other studies in India, Nigeria, and Tanzania, which showed that children under 24 months had a higher prevalence, ranging from 15.3% to 53.6%.^8,15,16^ This age group is at risk because of their immature immune systems and a high chance of colonic pathogens colonising the urinary tract system. Wearing nappies, as well as the possibility of having urinary tract malformation such as vesicle ureteric reflux, makes this age group susceptible to recurrent UTI.

UTIs can be caused by virtually any pathogen colonising the periurethral region, such as bacteria, viruses, and fungi. This study focused on bacterial causes of UTIs. We found a predominance of Gram-negative bacteria, with *E coli* and *K pneumoniae* being the most common pathogens causing UTI in children (prevalence of 46.2% and 30.8%, respectively). These findings are consistent with previous studies in India and Bangladesh, where *E coli* and *K pneumoniae* were predominant uropathogens.^5,16,17^ The only Gram-positive bacterium in this study was *S aureus*, reported in 1 urine sample, which might be due to contamination; however, *S aureus* in urine has been reported from other studies.^8,18^ In view of this finding, Gram-negative bacteria should be considered in children with UTI when selecting antibiotics for empiric treatments.

In this study, antimicrobial susceptibility to commonly used antibiotics for empiric treatment of UTI (aminoglyco-sides, ampicillin, penicillin, cephalosporin, co-trimoxazole, and quinolones) was low, particularly amongst the main bacterial isolates. Susceptibility was moderate to high for gentamicin, ciprofloxacin, ceftriaxone, ceftazidime, and nalidixic acid. However, we did identify amoxicillin-clavulanate and chloramphenicol as likely to be effective, with clindamycin having the lowest susceptibility in all organisms isolated. Similar sensitivity patterns have been observed in the United Kingdom by Bean and colleagues who found low susceptibility to ampicillin and co-trimoxazole, whereas susceptibility to nitrofurantoin, gentamicin, and ceftriaxone was high.^19^ These findings are also in agreement with studies done in Asia, the Middle East, other parts of Africa, and in previous work in Tanzania.^5,8,20,21,22^ Thus, with continue empiric treatment, UTI with resistance to antibiotics will become a global problem.

As a referral hospital, children admitted to our hospital are already being exposed to antibiotics that are overprescribed and widely available without prescription. Such practice is likely driving the increase in antibiotic resistance and lowers the chance of antibiotic choice. Exposure to antibiotics may play a role in lower culture-positive results in children exposed to antibiotics, as seen in this study, thus masking the situation. Therefore, clinicians and other health-care providers in lower-level health facilities should consider prescribing empiric antibiotics as per recommended national treatment guidelines, after performing simple screening tests like urine dipstick or microscopic examination, if available. Thus, public health awareness and education efforts may help the general population appreciate that antibiotics should be purchased only with a prescription after proper diagnosis is made. Health-care practitioners should prescribe antibiotics according to national guidelines; however, when unavailable, prescriptions for empiric antibiotics should be given only after excluding all other causes (eg, malaria and viral infection).

### Study Limitations

This study included all children with or without fever regardless of antibiotic exposure, which may underestimate the prevalence and aetiology of UTI. Moreover, caregivers of 36.3% of children previously exposed to antibiotics had no information about what kinds of antibiotics were used; it is therefore possible that this study underestimated the levels of antibiotic resistance.

## CONCLUSIONS AND RECOMMENDATION

UTIs are amongst the most common conditions affecting children admitted at KCMC, with a higher prevalence amongst infants and children younger than 24 months and females. The most common aetiological bacterial pathogens were *E coli* (46.2%) and *K pneumoniae* (30.8%), both of which showed low susceptibility to ampicillin, cotrimoxazole, and clindamycin. Antimicrobials, such as ciprofloxacin, ceftriaxone, and gentamicin are more likely to be successful for empiric treatment of UTIs. However, the susceptibility of the pathogens is still not high. Because susceptibility is not static, monitoring of antibiotics for UTI needs to be done continuously and guided with urine culture and antimicrobial sensitivity whenever possible.
